# Aorto-mitral intervalvular fibrosa pseudoaneurysm with mitral valve perforation after manouguian aortic root enlargement

**DOI:** 10.1093/ehjcr/ytag704

**Published:** 2026-09-17

**Authors:** Aby Dixon Pynadath, Tinoy Kizhakekuttu, Matthew Cox, Emmanuel Amulraj

**Affiliations:** Department of Anesthesiology, OSF HealthCare Saint Francis Medical Center, 530 NE Glen Oak Avenue, Peoria, IL 61637, USA; Department of Cardiology, OSF HealthCare Saint Francis Medical Center, 530 NE Glen Oak Avenue, Peoria, IL 61637, USA; Department of Cardiology, OSF HealthCare Saint Francis Medical Center, 530 NE Glen Oak Avenue, Peoria, IL 61637, USA; Department of Cardiovascular Surgery, OSF HealthCare Saint Francis Medical Center, 530 NE Glen Oak Avenue, Peoria, IL 61637, USA

## Summary

A 59-year-old patient developed heart failure symptoms five years after bioprosthetic surgical aortic valve replacement with Manouguian root enlargement. Multimodality imaging demonstrated an aorto-mitral intervalvular fibrosa pseudoaneurysm, suspected patch dehiscence, and a systolic jet from the left ventricular outflow tract to the left atrium. This case highlights the potential for delayed complications after Manouguian root enlargement, warranting careful long-term follow-up and evaluation.

## Case description

A 59-year-old patient with prior bioprosthetic valve replacement with Manouguian root enlargement for bicuspid aortic valve presented 5 years later with NYHA class III–IV symptoms.

Transthoracic and transoesophageal echocardiography demonstrated a pulsatile anechoic cavity adjacent to the aortic root and left ventricular outflow tract (LVOT), expanding in systole and collapsing in diastole (*Figure 1A* and *C*). Findings were consistent with a pseudoaneurysm near aorto-mitral curtain. A defect inferior to the enlargement patch was concerning for patch dehiscence (*Figure 1C*, Supplementary material online, *Video S1*). Colour Doppler demonstrated a systolic jet from the LVOT into the left atrium (LA), concerning for an LVOT-to-LA fistula or mitral valve perforation (*Figure 1B* and *D*). The prosthetic valve appeared tilted but well seated.

**Figure 1:**
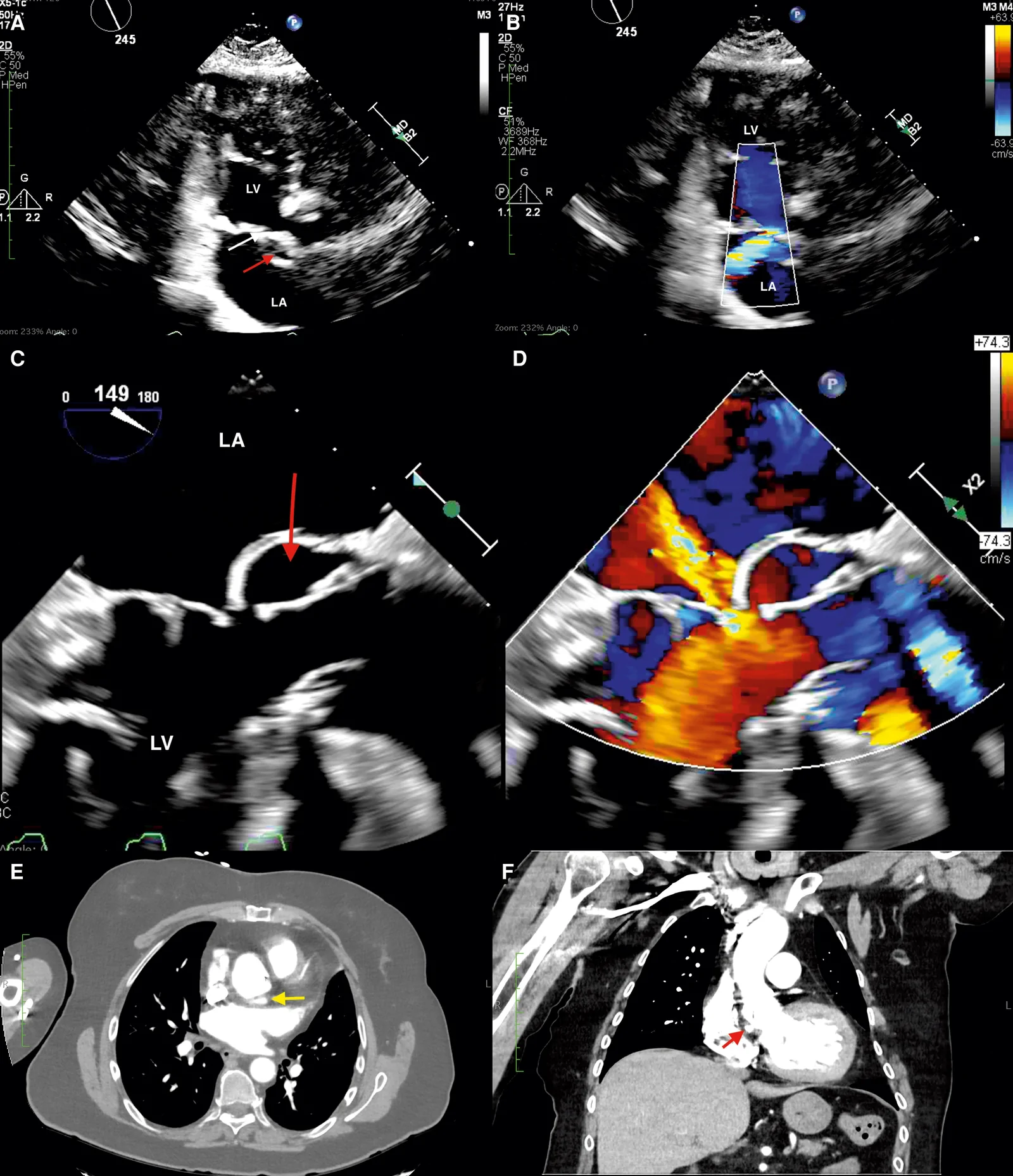
Multimodality imaging of an aorto-mitral intervalvular fibrosa pseudoaneurysm with left ventricular outflow tract–to–left atrial communication. (*A*) Transthoracic echocardiography demonstrates a pulsatile anechoic structure adjacent to the aortic root and left ventricular outflow tract (red arrow), with a defect at the base of the left ventricular outflow tract adjacent to the mitral valve (white arrow). (*B*) Transthoracic echocardiography with colour Doppler demonstrates a systolic jet from the left ventricular outflow tract into the left atrium. (*C*) Transoesophageal echocardiography demonstrates the pseudoaneurysm (red arrow), a defect inferior to the prior enlargement patch, and a second defect involving the left ventricular outflow tract–basal mitral leaflet region. (*D*) Transoesophageal echocardiography with colour Doppler demonstrates systolic flow from the left ventricular outflow tract into the left atrium. (*E*) Axial contrast-enhanced computed tomography demonstrates the contrast-filled outpouching adjacent to the aortic root (yellow arrow). (*F*) Coronal contrast-enhanced computed tomography further delineates the contrast-filled outpouching (red arrow).

Infective endocarditis was considered unlikely based on negative blood cultures, serology, PET CT, and microbial cell-free DNA testing. Cardiac CT demonstrated a contrast-filled pseudoaneurysm at the left coronary sinus between the aortic root and LA, largest in systole (*Figure 1E* and *F*).

Because the patient was initially reluctant to undergo redo surgery, echocardiographic and CT surveillance was pursued every 6 months. At 1 year, a slight increase in perivalvular contrast on CT along with worsening symptoms prompted surgery. Intraoperatively, the communication arose from perforation of the basal anterior mitral leaflet. Recovery was uneventful, and the patient did well on follow-up.

Manouguian aortic root enlargement extends the aortotomy across the commissure between the left and non-coronary cusps, through the aorto-mitral intervalvular fibrosa, to the base of the anterior mitral leaflet, with patch reconstruction of the resulting defect.^1^ This fibrous, relatively avascular region is vulnerable to delayed structural breakdown, predisposing to pseudoaneurysm formation and fistulous communication with the LA.

Pseudoaneurysm and mitral valve perforation are late complications of Manouguian root enlargement. Abnormal systolic flow near the prosthetic aortic root in a post-enlargement patient warrants evaluation for both.^2^

## Supplementary Material

ytag704_Supplementary_Data

## Data Availability

The data underlying this article will be shared on reasonable request to the corresponding author.

## Supplementary material

**Consent:** The authors confirm that written consent for the submission and publication of this case report, including images and associated text, was obtained from the patient in accordance with the COPE guidelines.
